# Synthesis and Radioprotective Activity of Mitochondria Targeted Dihydropyridines In Vitro

**DOI:** 10.3390/ijms18112233

**Published:** 2017-10-25

**Authors:** Yurui Zhang, Junying Wang, Yuanyuan Li, Feng Wang, Fujun Yang, Wenqing Xu

**Affiliations:** 1 Tianjin Key Laboratory of Molecular Nuclear Medicine, Institute of Radiation Medicine, Chinese Academy of Medical Sciences and Peking Union Medical College, Tianjin 300192, China; tianchuanruirui@163.com (Y.Z.); yuanyuanli0929@163.com (Y.L.); fjyang32@126.com (F.Y.); 2 Department of Physics, School of Sciences and Tianjin Collaborative Innovation Center of Chemical Science and Engineering, Tianjin University, Tianjin 300350, China; wangjunying@tju.edu.cn; 3 Department of Statistics, Tianjin University of Finance and Economics, Tianjin 300222, China; kathrinewang95@outlook.com

**Keywords:** reactive oxygen species (ROS), oxidative stress, mitochondrial targeted, radiation damage

## Abstract

The radiation-induced damage to mitochondrial oxidative respiratory chain could lead to generating of superoxide anions (O^2−^) and secondary reactive oxygen species (ROS), which are the major resources of continuous ROS production after radiation. Scavenging radiation-induced ROS effectively can help mitochondria to maintain their physiological function and relief cells from oxidative stress. Dihydropyridines (DHPs) are biomimetic hydrogen sources that could protect cells against radiation damage. In this study, we designed and synthetized three novel mitochondrial-targeted dihydropyridines (Mito-DHPs) that utilize the mitochondrial membrane potential to enter the organelle and scavenge ROS. MitoTracker confirmed Mito-DHPs accumulation in mitochondria, and the DCFH-DA assay demonstrated effective ROS scavenging activity. In addition, the γ-H2AX and comet assay demonstrated the ability of Mito-DHPs to protect against both radiation and ROS-induced DNA strand breaks. Furthermore, Mito-DHP1 proved to be non-toxic and displayed significant radioprotection activity (*p* < 0.05) in vitro. Mito-DHPs are therefore promising antioxidants that could penetrate the membrane of mitochondria, scavenge excessive ROS, and protect cells against radiation-induced oxidative damage.

## 1. Introduction

Radiation technology is increasingly applied in physical and medical fields such as nuclear energy and radiotherapy, which is still a dominating way for cancer treatment [1]. Radiation-induced damage is a tremendous potential threat to human body. Although screening and synthesis of radio-protective compounds are well established, but few effective compounds are currently available except the amino-thiols [2,3,4], polyphenols [5], and growth factor [6,7], all of which also have undesirable side effects limited their clinical applications [7]. Therefore, it is necessary to develop substances that protect cells and tissues against radiation-induced injury.

Exposure to ionizing radiation results in oxidative stress, DNA damage, aging, chronic disease, cancers, and death. Radiation-induced damage can be divided into direct and indirect damage [8]. Direct damage causes damage of DNA and proteins under the high energy rays, while indirect damage is caused by excessive reactive oxidative species (ROS). Actually, the intracellular ROS burst was mostly ascribed to radiation-induced mitochondria damage [9]. Mitochondria are responsible for cellular oxidative respiration through the process of oxidative phosphorylation [10,11], where the potential damaging free radical superoxide anion was constantly produced [12,13]. Mitochondrial disturbance could lead enormous free radicals released towards cytoplasm, trigger chain reactions, and ultimately turn the cell into pathological conditions [14]. Therefore, once mitochondrial dysfunction happens, the excessive ROS—such as superoxide, hydrogen peroxide, and hydroxyl radical—with high chemical reactivity, could destroy cell structures through interacting with functional molecules such as fatty acids, nucleic acids, and proteins and ultimately disrupting cell functions [15]. Ionizing radiation not only causes mitochondrial dysfunction by increasing oxidative stress [9,16], but also causes apoptosis and autophagy through ROS-mediated regulation of various pathways, because mitochondria are involved in signaling the oxidative state to the nucleus [17]. Furthermore, evidence suggests that nuclear DNA is the primary target of ionizing radiation, since ROS reaction with DNA appears to lead to genomic instability. Currently, various naturally occurring antioxidants could scavenge ROS, but they may have unacceptable toxicity at the same time [18,19]. Therefore, finding an effective antioxidant with low cell toxicities is still an urgent problem to be solved.

1,4-dihydropyridines (DHPs) are a large, structurally diverse group of Ca^2+^ antagonists [20]. They have excellent antioxidant properties and ROS scavenging abilities [14]. However, despite their powerful free-radical scavenging capabilities, DHPs cannot be accumulated to mitochondria largely without the positive charged lipophilic structure. In this study, we designed and synthesized three novel DHPs derivatives that included a positively charged triphenylphosphonium (TPP) group [21]. These novel cationic compounds were able to use the membrane potential, which is critical for ATP synthesis and maintaining mitochondrial function, to cross the mitochondrial membrane in order to scavenge ROS and protect against radiation-induced mitochondrial oxidative damage.

## 2. Results

### 2.1. Cellular Distributions of Mito-DHPs

**Figure 1:**
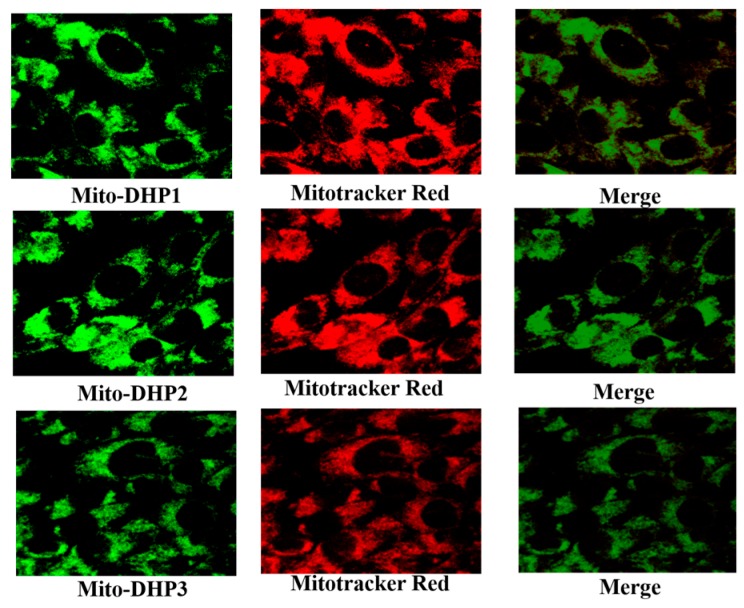
Accumulation of Mito-DHPs in the mitochondria of CHO-K1 cells. Fluorescence images of cells incubated with 10^−6^ mol/L Mito-DHPs for 2 h and stained with Mitotracker Red (200 nM, 15 min). Merged images show co-localization of Mito-DHP and Mitotracker Red which suggested their successful targeting to mitochondria.

The commercially available Mitotracker Red probe [22] contains a mildly thiol-reactive chloromethyl moiety for labelling mitochondria [23], and we used it to investigate the cellular distribution of the three new structures, along with Mito-DHPs intrinsic green fluorescence. The overlapping merged image in Figure 1 suggested that the localization of two labelling molecules were in good agreement, which confirmed that all three compounds were able to target mitochondria.

### 2.2. Reactive Oxygen Species (ROS) Detection

**Figure 2:**
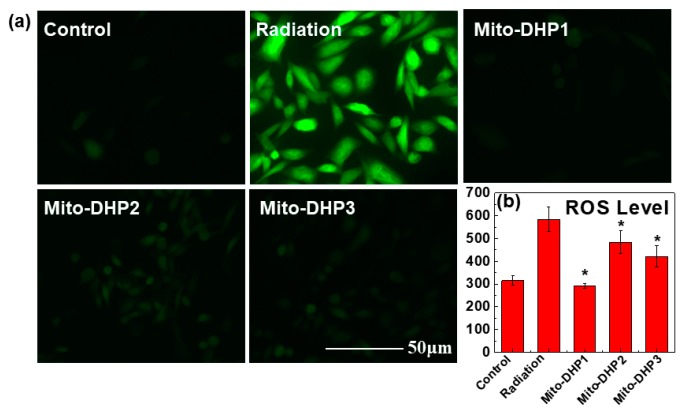
ROS detection using the DCFH-DA agent. (**a**) Fluorescence images of CHO-K1 cells from different groups following incubation with DCFH-DA for 30 min; (**b**) ROS level in Mito-DHPs treated and untreated cells at 24 h after 3 Gy radiation. (* *p* < 0.05 compared with radiation group).

The dichloro-dihydro-fluorescein diacetate (DCFH-DA) assay was used to detect intracellular ROS level after radiation. As shown in Figure 2a, the radiation group had a strong fluorescent signal after incubation with DCFH-DA. Compared with radiation group, Mito-DHPs pretreated groups showed weak fluorescence. Among which Mito-DHP1 displayed similar ROS level to cells in control group. Data obtained from the Infinite F200 multimode plate reader in Figure 2b also proved that Mito-DHP1, Mito-DHP2, and Mito-DHP3 treated groups displayed reduced accumulation of ROS both to CHO and HeLa cells (Figure S1). These results suggest the Mito-DHPs were effective intracellular ROS scavengers that prevented ROS from accumulation and Mito-DHP1 was the most effective radio-protective compound.

### 2.3. Detection of Histone H2AX (γ-H2AX)

**Figure 3:**
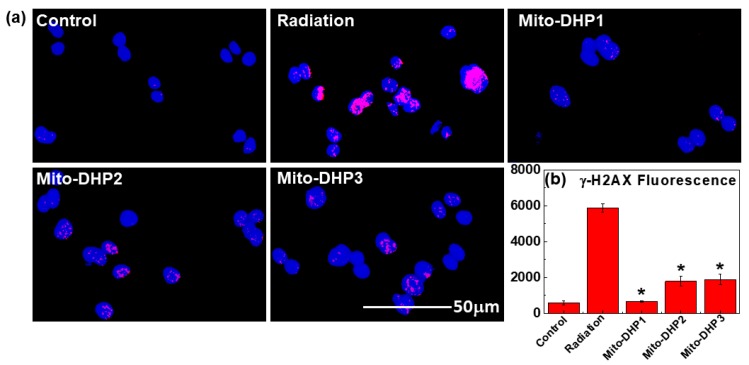
Determination of phosphorylated histone H2AX (γ-H2AX) in CHO-K1 cells at 1 h after γ radiation. (**a**) Images of cells obtained during the immunofluorescence assay showed γ-H2AX foci in different treatment groups; (**b**) γ-H2AX foci fluorescence of control, radiation and Mito-DHPs treated groups after 3 Gy radiation. (* *p* < 0.05 compared with radiation group).

Histone H2AX (γ-H2AX) is a marker of DNA double strand breaks (DSBs) [24], and we used this marker in an immunofluorescence approach to investigate irradiation-induced DSBs [25]. Images in Figure 3a showed γ-H2AX signals (pink spots) on the DAPI background, corresponding data are presented in Figure 3b. Compared with the radiation group, Mito-DHP1 pretreated group showed decreased γ-H2AX foci fluorescence to 654, suggested higher radioprotection than Mito-DHP2 and Mito-DHP3, which was 1781 and 1885 respectively (Figure 3b). The results suggested that our compounds could effectively reduce γ-H2AX in radiated cells, therefore scavenging radiation-induced ROS and preventing DNA damage.

### 2.4. Comet Assay

**Figure 4:**
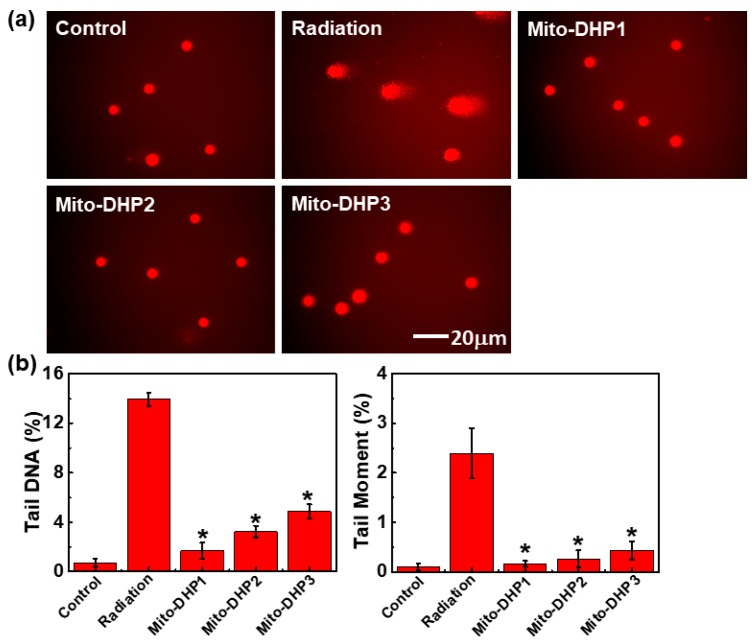
Comet assay demonstrated DNA damage in CHO-K1 cells after 3Gy γ-radiation. (**a**) Comet images from Mito-DHPs treated and untreated groups. Cells from radiation groups displayed clear tail trailing, whereas tail trailing in Mito-DHPs-treated groups was at a similar level compared with the non-irradiated control group; (**b**) Tail DNA (%) and Tail Moment (%) representing nuclear DNA damage following a 3 Gy radiation dose. These parameters are markedly reduced in cells pretreated with Mito-DHPs (* *p* < 0.05).

The Comet assay is commonly used method for evaluating DNA strand breaks, and we evaluated olive tail moment (OTM) and tail DNA (TD) to further assess radiation-induced DNA damage [26]. OTM and TD signify the length of DNA in the comet tail relative to total DNA. Images of nuclear DNA was presented in Figure 4a, radiation group showed obvious DNA tail after radiation. However, nuclear DNA in compounds treated groups were intact post radiation, comparable with normal cell nuclear DNA in control group. The OTM and TD analysis results from Figure 4b showed a significant decrease in mean OTM and TD in Mito-DHPs-treated cells (compared with the radiation group), which indicated that Mito-DHPs have strong radio-protective abilities. All three compounds protected DNA against radiation-induced damage. Again, Mito-DHP1 provided higher radioprotection than Mito-DHP2 and Mito-DHP3 which corresponded well with our previous results.

## 3. Discussion

When exposed to ionizing radiation, cells produce ROS that cause oxidative stress and attack bio-macromolecules—such as lipids, nucleic acids, and proteins—which lead lethal consequences to human body [27,28]. At beginning, ROS are formed from radiation-induced hydrolysis of water, which generates hydroxyl radicals (•OH) as the primary ROS [29]. •OH radicals exist only for a few minutes [17,30], but during that time they react with other neutral molecules into radicals in a destructive chain reaction. Intracellular ROS attack enzymes involved in oxidative phosphorylation and the electron transport chain (ETC) in mitochondria [31]. Peroxidation of mitochondria membrane lipids disrupts enzyme activity and promotes leakage of O^2−^ from the ETC, which is subsequently converted to H_2_O_2_ or hydroxyl radicals through the Fenton reaction as secondary free radicals, leading to oxidative stress and damage to nuclear DNA [32]. Levels of secondary ROS in mitochondria are increased after irradiation, causing wide-ranging and persistent oxidative stress [16].

Mitochondria are known to play an important role in mediating radiation-induced cell damage [33,34,35]. Considering the important function of mitochondria, we designed and synthesized a series of dihydropyridines derivative (Mito-DHPs) by incorporating a positively charged triphenylphosphonium moiety that could target the molecule to mitochondria by facilitating its passage across the mitochondrial double membranes system. Based on the Nernst equation, the uptake of Mito-DHPs into mitochondria can increase 10-fold for every 60 mV of membrane potential [36]. Mito-DHPs are accumulated in the cytoplasm with the help of membrane potential (Δψ), and reach a concentration that is 5–10-fold higher than outside the cell. Mito-DHPs also could reach a further 500–1000-fold in mitochondria since the mitochondrial membrane potential is further increased.

In our study, Mito-DHP1, Mito-DHP2, and Mito-DHP3 could effectively protect cells from radiation-induced damage due to their 1,4-dihydropyridine structure, the parent nucleus of NAD(P)H [37]. On one hand, 1,4-dihydropyridine is a good hydrogen donor [38] through its NH and C–4 H groups reacting with oxidants and free radicals. Excessive harmful free radicals in organisms are responsible for developing severe diseases including neurodegenerative disease, aging, obesity, cancer, and metabolic as well as cardiovascular disorders [39,40,41,42,43]. Although effective antioxidants have been investigated for decades, most existing antioxidants such as polyphenol or amino-thiol compounds are unfortunately highly toxic [44,45]. Amino-thiol compounds can influence the concentration of metal ions such as Ca^2+^ and cause hypotension [46], whereas polyphenol compounds are metabolized to quinone, which is toxic to DNA [47,48,49]. On the other hand, 1,4-dihydropyridine structure represents the active part of reduced coenzyme—NAD(P)H—which is important modulators of various enzymatic redox reactions and are involved in electron transfer. The study showed thiyl and phenoxyl free radicals could transform into stable structure through one-electron-transfer reactions with NAD(P)H [50]. It is also important to maintain a high level of glutathione (GSH), which can help to prevent oxidative stress. Besides, Mito-DHPs presented lower cell toxicity in CHO-K1 cells (Figure S2) and HeLa cells (Figure S3), Mito-DHP1 notably displayed minimum toxicity to CHO cells (Figure S2). To investigate whether the compounds could protect cells from radiation and to determine an appropriate dose for administration, five doses (10^−4^ to 10^−8^ mol/L) were tested for three compounds and cell survival was monitored by measuring their ability to absorb Neutral Red (NR) dye (Figures S4 and S5), and 10^−6^ mol/L was considered as the optimum dosage.

These three compounds protect DNA from radiation-induced damages, and may influence initiation and propagation of free radicals. As the results show, Mito-DHP1, Mito-DHP2, and Mito-DHP3 are effective ROS scavengers that are supposed to donate an H atom to free radicals converting into neutral molecules. Mito-DHP1 is superior to Mito-DHP2 and Mito-DHP3 in protecting cells against oxidative damage. Mito-DHP2 and Mito-DHP3 (4-phenyl-1,4-dihydropyridine and 4-thiophenyl-1,4-dihydropyridine, respectively) exhibited reasonable lower antioxidative activity compared with Mito-DHP1 (4-unsubstituted-1,4-dihydropyridine). This finding was consistent with the results of other studies that substituents at the 4 position appear to diminish antioxidant activity [51]. Mito-DHP1 not only exhibited excellent radioprotection activity, but also displayed lower toxicity than the other two compounds. This phenomenon could be ascribed to two aspects, firstly, electron donating and withdrawing effects appeared to diminish the antioxidant activity of the 4-substituted-1,4-dihydropyridine compounds. Secondly, compared with NAD(P)H, 4-substituted-1,4-dihydropyridines may show a further stereospecific blockade at the 4 position which presents a poor catalytic performance.

In general, all three compounds designed in this study were able to effectively scavenge radiation-induced ROS in mitochondria, and help to maintain normal mitochondrial function and protect cells from oxidative stress. Most importantly, all three compounds could effectively protect DNA against radiation-induced break. In all experiments, Mito-DHP1 was markedly more effective than Mito-DHP2 and Mito-DHP3.

## 4. Materials and Methods

### 4.1. Chemistry

**Scheme 1:**
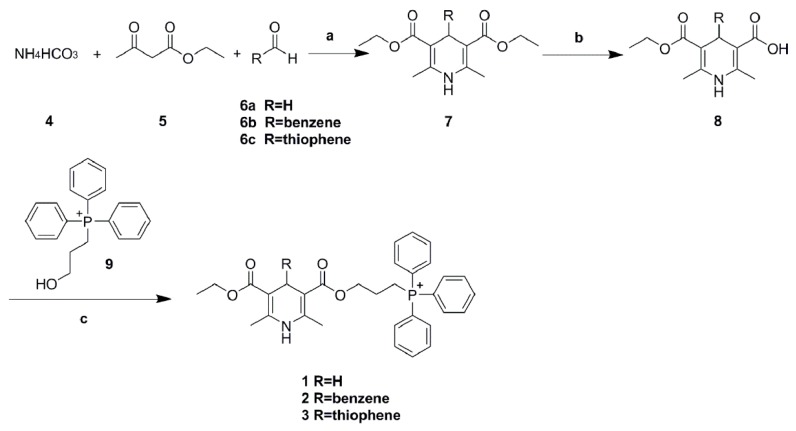
Reagents and conditions: (**a**) CH_3_CH_2_OH, 80 °C, under nitrogen, overnight; (**b**) NaOH, CH_3_OH, 1 M HCl, 80 °C, 6 h; (**c**) EDCI, HOBT, Et_3_N, CH_2_Cl_2_, room temperature, overnight.

**Scheme 2:**
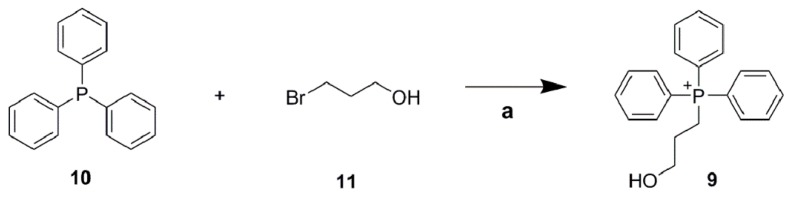
Reagents and conditions: (**a**) DMF, 100 °C, overnight.

**Figure 5:**
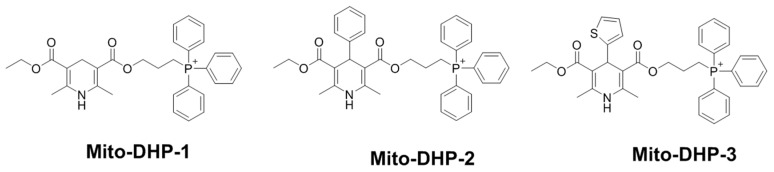
Representative targeted compounds.

Target compounds **1**–**3** were synthesized as shown in Scheme 1. In a similar procedure to that reported previously in the literature [52], commercially available NH_4_HCO_3_, **4**, ethyl 3-oxobutanoate, **5**, and aldehydes, **6**a–**6**c were condensed in ethanol to form the key intermediate 1,4-dihydropyridines, **7**a–**7**c, which was then hydrolyzed under basic condition to give acid **8**a–**8**c. Compound **8**a–**8**c was reacted with (3-hydroxypropyl) triphenylphosphonium, **9**, which was synthesized from triphenylphosphine, **10**, and 3-bromopropan-1-ol, **11**, (Scheme 2), to give the final target compounds **1**–**3**, respectively named Mito-DHP1, Mito-DHP2, Mito-DHP3 (Figure 5).

### 4.2. Biological Evaluation

Cell culture and radiation—the Chinese hamster ovary (CHO)-K1 and HeLa cell line was cultured at 37 °C under a humidified atmosphere with 5% CO^2^ in Dulbecco’s High Glucose Modified Eagles Medium (DMEM/HIGH GLUCOSE; HyClone, Logan, UT, USA), supplemented with penicillin (100 units/mL), streptomycin (100 mg/mL; Gibco, Carlsbad, CA, USA), and 10% fetal bovine serum (Gibco). Cells were grown in 6- or 96-well culture plates with 4 × 10^3^ and 1 × 10^5^ cells per well and incubated overnight. Before irradiation, the culture medium was replaced by fresh medium with Mito-DHPs (1 μM), and kept incubating for 30 min. Cell irradiation was performed at the Institute of Radiation Medicine, Chinese Academy of Medical Sciences, with a Cs^137^ γ-radiation source at a power of 0.98 Gy/min, 3 Gy for final dose.

Intracellular accumulation of Mito-DHPs: CHO-K1 cells were incubated with 10 μM Mito-DHPs at 37 °C for 1 h. Culture medium was then removed from the 6-well culture plates, and pre-warmed (37 °C) Mitotracker Red (20 nM) staining solution was added. Cells were incubated for 20 min at 37 °C. After staining, all wells were washed three times with fresh pre-warmed PBS (37 °C). Mito-DHPs accumulations in cells were visualized using a Leica confocal microscope.

ROS detection: CHO-K1 and HeLa cells were incubated with 1 mL of 5 μM 2,7-dichlordihydrofluorescein diacetate (DCFH-DA) for 20 min at 37 °C, 24 h post radiation. Cells were washed three times with PBS, and the fluorescence intensity was measured at 488 nm with an Infinite F200 multimode plate reader or a flow cytometry.

Detection of γ-H2AX: Histone H2AX (γ-H2AX) was used to monitor DNA double strand breaks (DSBs) and was calculated by immunofluorescent foci. CHO cells were incubated at 37 °C for 1 h after radiation, and then fixed with 4% paraformaldehyde for 15 min, washed with PBS, and treated with 0.2% Triton-X 100 for 15 min. After PBS washing, cells were blocked with 10% goat serum for 1 h, and incubated with rabbit polyclonal γ-H2AX (phospho S139) primary antibody (dilution 1:1000; cat. no. ab2893; Abcam, Cambridge, MA, USA) at 4 °C overnight. Washing the primary antibody three washes with PBS, cells were incubated with goat anti-rabbit fluorescent secondary antibody (dilution 1:2000; cat. no. ab6939; Abcam). Nuclei were counterstained with DAPI (cat. no. C0065, Solarbio, Beijing, China). Images of cells were obtained using an AMG EVOS fluorescence microscope. Image Pro Plus 6.0 software was used to count foci in each pictures.

Comet assay: After radiation, cells were mixed with 70% low-melting-point agarose (0.6%) and placed on microscope slides covered with agarose (0.8%). Slides were submerged in freshly prepared cold lysis buffer containing 2.5 M NaCl, 100 mM Na_2_EDTA, 10 mM Tris-HCl, 10% DMSO, and 1% Triton X-100 for 2.5 h. Slides were then immersed in a horizontal gel electrophoresis unit filled with cold electrophoresis buffer containing 1 mM Na_2_EDTA and 300 mM NaOH for 30 min. Electrophoresis was conducted at 30 V for 20 min. Slides were neutralized after electrophoresis with Tris-HCl (pH = 7) and stained with ethidium bromide (2 μg/mL). Slides were examined using a Nikon fluorescence microscope and DNA damage was estimated using the Comet Assay Software Project (CASP).

## Supplementary Materials

Supplementary materials can be found at www.mdpi.com/1422-0067/18/11/2233/s1.
