# Supplementary material for: Synthesis and Radioprotective Activity of Mitochondria Targeted Dihydropyridines In Vitro
Source: Int J Mol Sci. 2017 Oct 25;18(11):2233. doi: 10.3390/ijms18112233 (PMC5713203; doi:10.3390/ijms18112233)
Supplement: Supplementary file 1 [file ijms-18-02233-s001.pdf]

Supporting Information

Synthesis and Radioprotective Activity of Mitochondria Targeted Dihydropyridines In Vitro

Yu-Rui Zhang[1], Jun-Ying Wang[2], Yuan-Yuan Li[1], Feng Wang[3], Fu-Jun Yang[1], and Wen-Qing Xu [1,*]

[1] Tianjin Key Laboratory of Molecular Nuclear Medicine, Institute of Radiation Medicine, Chinese Academy of Medical Sciences and Peking Union Medical College, Tianjin, 300192, China; tianchuanruirui@163.com
[2] Department of Physics, School of Sciences and Tianjin Collaborative Innovation Center of Chemical Science and Engineering, Tianjin University, Tianjin 300350, China; wangjunying@tju.edu.com
[3] Department of Statistics, Tianjin University of Finance and Economics, Tianjin 300222, China; 1171760063@qq.com
* Correspondence: xuwenqing@irm-cams.ac.cn Tel.: +(022) 85683049

1. Chemistry

All evaporations were carried out in vacuo with a rotary evaporator (BuChi R-210). Analytical samples were dried in a DZF-6020 vacuum (0.2 mm Hg) drying oven over P_2O_5. ^1H NMR spectra were recorded on a Varian Inova 500MHz NMR spectrometer. Chemical shift values are expressed in parts per million (ppm) relative to tetramethylsilane as an internal standard; s = singlet, d = doublet, t = triplet, q = quartet, m = multiplet, br = broad singlet. The relative integrals of peak areas agreed with those expected for the assigned structures. ^{13}C NMR spectra were recorded on a Varian Inova 500MHz NMR spectrometer. Mass spectra (MS) were determined using a Waters 3100 mass spectrometer. Thin-layer chromatography (TLC) was carried out on silica gel GF254 plates, and spots were visualized under 254 and 365 nm illumination. Column chromatography was performed on 300–400 mesh silica gel. Melting points were determined on a digital melting point apparatus and remained uncorrected. All solvents and chemicals were purchased from Aldrich Chemical Co. and Tokyo Chemicals Industry Co. Ltd. and were used as received. The purities of all assayed compounds were determined by analytical HPLC and found to be at least 95% pure unless otherwise specified. Analytical HPLC was performed using a waters 2695 apparatus equipped with a photodiode array detector.

1.1. General Procedure for the Synthesis of Intermediates 7a–7c

A mixture of **6a–6c,** NH_4HCO_3 **4,** (7.9 g, 0.1 mol), ethyl 3-oxobutanoate **5**, (26 g, 0.2 mol), and ethanol (30 mL) was added to a 100 mL round-bottomed flask equipped with a magnetic stirrer and refluxed under nitrogen for 20 h until a yellow precipitate appeared. The suspension was cooled to room temperature, filtered, washed with cold ethanol (2 × 20 mL), and dried to afford **7a–7c** as a yellow powder.

1.1.1. *Diethyl 2,6-dimethyl-1,4-dihydropyridine-3,5-dicarboxylate* (7a)

Compound **7a** was prepared using the general method described for the preparation of **7a–7c** from formaldehyde **6a,** (3.0 g, 0.1 mol) to give **7a** as a yellow powder (20.32 g, yield= 80.3%); mp =182–183.8°C (lit. mp = 182–183°C); ^1H NMR (500 MHz, DMSO-d_6), δ = 8.28 (s, 1H, -N**H**), 4.04 (q, *J* = 7.1 Hz, 4H, -C**H**$_2$CH$_3$), 3.09 (s, 2H, -C**H**$_2$-), 2.09 (s, 6H, -C**H**$_3$), 1.17 (t, *J* = 7.1 Hz, 6H, -CH$_2$C**H**$_3$); ESI-MS (m/z) = 252.5[M-H]$^+$.

1.1.2. Diethyl 2,6-dimethyl-4-phenyl-1,4-dihydropyridine-3,5-dicarboxylate (7b)

Compound **7b** was prepared using the general method described for the preparation of **7a–7c** from benzaldehyde **6b,** (10.6 g, 0.1 mol) to give **7b** as a white powder (20.62 g, yield=62.7%); mp =155.8–156.9°C (lit. mp = 156–157°C), ^{1}H NMR (500 MHz, DMSO-d_6), δ = 8.80 (s, 1H, -N**H**), 7.20–7.05 (m, 5H, benzene), 4.84 (s, 1H, -C**H**), 3.96 (dd, *J* = 9.2, 7.1 Hz, 4H, -C**H**$_2$CH$_3$), 2.24 (s, 6H, -C**H**$_3$), 1.11 (t, *J* = 7.1 Hz, 6H, -CH$_2$C**H**$_3$); ESI-MS (m/z) = 328.5[M-H]$^+$.

1.1.3. Diethyl 2,6-dimethyl-4-(thiophen-2-yl)-1,4-dihydropyridine-3,5-dicarboxylate (7c)

Compound **7c** was prepared using the general method described for the preparation of **7a–7c** from thiophene-2-carbaldehyde **6c,** (11.2 g, 0.1 mol) to give **7c** as a white powder (24.32 g, yield=72.5%); mp = 156.2–159.4°C (lit. mp = 156–159°C); ^{1}H NMR (500 MHz, DMSO-d_6), δ = 9.01 (s, 1H, -N**H**), 7.18 (d, *J* = 5.1 Hz, 1H, thiophene), 6.82 (dd, *J* = 5.0, 3.5 Hz, 1H, thiophene), 6.64 (d, *J* = 3.4 Hz, 1H, thiophene), 5.15 (s, 1H, -C**H**), 4.12–3.97 (m, 4H, -C**H**$_2$CH$_3$), 2.25 (s, 6H, -C**H**$_3$), 1.16 (t, *J* = 7.1 Hz, 6H, -CH$_2$C**H**$_3$); ESI-MS (m/z) = 334.5 [M-H]$^+$.

1.2. Procedure for the Synthesis of Intermediate 9

Compound **9** was synthesized from a mixture of 3-bromo-1-propanol **11** (0.5 g, 3.6 mmol) and triphenylphosphine **10** (9.4 g, 35.9 mmol) in DMF. The mixture was gently stirred at 100°C for 10 h, poured into a fritted funnel, washed with warm dimethylformamide (DMF; 3 × 50 mL), dichloromethane (CH_2Cl_2; 3 × 50 mL), and removal of the solvent under reduced pressure gave a white solid. Then, the dried solid solvent was removed under pressure at 40°C overnight to give a white solid (0.9 g, 2.2 mmol, yield=62%). ^{1}H NMR (500 MHz, DMSO-d_6), δ = 7.92–7.72 (m, 15H, -P$^+$**Ph**$_3$), δ = 4.86 (t, J = 5.3 Hz, 1H, -O**H**), δ = 3.60–3.53 (m, 2H, -C**H**$_2$-P$^+$Ph$_3$), δ = 3.51 (dd, J= 11.3, 5.5Hz, 2H, -C**H**$_2$-OH), δ = 1.70–1.60 (m, 2H, -CH$_2$-C**H**$_2$-CH$_2$). ESI-MS (m/z) = 321.3 ($C_{21}H_{22}OP^+$).

1.3. General Procedure for the Synthesis of Intermediates 8a–8c

A mixture of **7a–7c,** 30 mL CH_3OH， 30 mL H_2O and NaOH (1.6 g, 0.04 mol) was added to a 100 mL round-bottomed flask equipped with a magnetic stirrer and refluxed with stirring for 5 h. TLC confirmed the disappearance of starting material (Rf = 0.67), and after completion of the reaction only one major spot was present at the origin (Petroleum ether/Ethyl acetate = 1:1). The solution was cooled in an ice bath and the pH was adjusted to 2.5 by dropwise addition of 3 M HCl. The resulting suspension was filtered and dried under vacuum using P_2O_5 to afford **8a–8c** as a white powder.

1.3.1. 5-(ethoxycarbonyl)-2,6-dimethyl-1,4-dihydropyridine-3-carboxylic acid (8a)

Compound **8a** was prepared using the general method described for the preparation of **8a–8c** from **7a** (5.06 g, 20 mmol) to give **8a** as a yellow powder (1.52g， yield=67.6%); mp = 191.1–192.6°C. ^{1}H NMR (500 MHz, DMSO-d_6), δ 11.72 (br, 1H, -COO**H**), 8.23 (s, 1H,-N**H**), 4.02 (q, *J* = 7.1 Hz, 2H, -C**H**$_2$CH$_3$), 3.07 (s, 2H, -C**H**$_2$-), 2.10 (s, 6H, -C**H**$_3$), 1.12 (t, 3H, -CH$_2$C**H**$_3$); ESI-MS (m/z) = 224.4 [M-H]$^+$.

1.3.2. 5-(ethoxycarbonyl)-2,6-dimethyl-4-phenyl-1,4-dihydropyridine-3-carboxylic acid (8b)

Compound **8b** was prepared using the general method described for the preparation of **8a–8c** from **7b** (6.5 g, 20 mmol) to give **8b** as white powder (1.45g, yield=24.3%); mp =192.1–193.6°C (lit. mp = 192–194°C). ^{1}H NMR (500 MHz, DMSO-d_6):, δ = 11.66 (br, 1H, -COO**H**), 8.75 (s, 1H, -N**H**), 7.15 (dd, *J* = 21.9, 7.1 Hz, 5H, benzene), 4.84 (s, 1H, -C**H**-), 4.01–3.93 (m, 2H, -C**H**$_2$CH$_3$), 2.24 (d, *J* = 2.6 Hz, 6H, -C**H**$_3$), 1.13 (t, *J* = 7.1 Hz, 3H, -CH$_2$C**H**$_3$); ESI-MS (m/z) = 300.5 [M-H]$^+$.

1.3.3. 5-(ethoxycarbonyl)-2,6-dimethyl-4-(thiophen-2-yl)-1,4-dihydropyridine-3-carboxylic acid (8c)

Compound **8a** was prepared using the general method described for the preparation of **8a–8c** from **7c** (6.70 g, 20 mmol) to give **8c** as a white powder (2.37g, yield=38.6%); mp =191–193.3°C. ^{1}H NMR (500 MHz, DMSO-*d*$_6$), δ 11.82 (br, 1H, -COO**H**), 8.94 (s, 1H, -N**H**), 7.17 (d, *J* = 4.4 Hz, 1H, thiophene), 6.82 (dd, *J* = 4.9, 3.6 Hz, 1H, thiophene), 6.64 (d, *J* = 3.3 Hz, 1H, thiophene), 5.13 (s, 1H,-C**H**-), 4.10–4.02 (m, 2H, -C**H**$_2$CH$_3$), 2.25 (d, *J* = 2.0 Hz, 6H, -C**H**$_3$), 1.18 (t, *J* = 7.1 Hz, 3H, -CH$_2$C**H**$_3$); ESI-MS (m/z) = 306.4 [M-H]$^+$.

1.4. General Procedure for the Synthesis of Target Compounds 1–3

A mixture of **8a–8c** (1.0 mmol), HOBt (162 mg, 1.2 mmol) and anhydrous DMF (15 mL) was added to a 100 mL round-bottomed flask equipped with a magnetic stirrer. After 2 h, EDCI (230 mg, 1.2 mmol), (3-hydroxypropyl)triphenylphosphonium (514 mg, 1 mmol), and Et$_3$N (202 mg, 2 mmol) were added and the reaction was stirred at room temperature overnight. After evaporation of solvent under reduced pressure, NaHCO$_3$ solution (20 mL) was added and the precipitate was filtered and purified by chromatography on silica gel to give the target compounds, which were dried in vacuum to afford **1–3** as white/yellow powders.

1.4.1. (3-((5-(ethoxycarbonyl)-2,6-dimethyl-1,4-dihydropyridine-3-carbonyl)oxy)propyl)triphenylphosphonium (1)

Compound **1** was prepared using the general method described for the preparation of 1-3 from 8a (450 mg, 2.0 mmol) to give 1 as a yellow powder (390 mg, yield=36.9%); ^{1}H NMR (500 MHz, DMSO-*d*$_6$): δ= 8.35 (d, *J* = 9.5 Hz, 1H, -N**H**-), 7.92 – 7.76 (m, 15H, -P**Ph**$_3$), 4.11 (t, *J* = 6.0 Hz, 2H, -C**H**$_2$-CH$_2$-CH$_2$-PPh$_3$), 4.12 – 3.97 (m, 2H, -C**H**$_2$CH$_3$), 3.15 (s, 2H, -C**H**$_2$-CH-CH-NH-), 2.78 – 2.65 (m, 2H, -CH$_2$-CH$_2$-C**H**$_2$-PPh$_3$), 2.10 (s, 3H, -C**H**$_3$), 2.08 (s, 3H, -C**H**$_3$), 1.85 (m, *J* = 14.6, 7.1 Hz, 2H, -CH$_2$-C**H**$_2$-CH$_2$-), 1.09 (t, *J* = 7.1 Hz, 3H, CH$_2$C**H**$_3$). ^{13}C NMR (500 MHz, CDCl$_3$): δ = 19.09, 19.18, 24.94, 25.11, 50.87, 52.47, 97.47, 98.46, 117.30, 118.16, 130.60, 130.65, 130.73, 130.77, 133.57, 133.67, 146.61, 147.84, 167.48, 168.56. ESI-MS (m/z) = 528.3 [M]$^+$.

1.4.2. (3-((5-(ethoxycarbonyl)-2,6-dimethyl-4-phenyl-1,4-dihydropyridine-3-carbonyl)oxy)propyl)triphenylphosphonium (2)

Compound 2 was prepared using the general method described for the preparation of **1-3** from **8b** (600 mg, 2.0 mmol) to give **2** as a yellow powder (640 mg, yield=52.9%); ^{1}H NMR (500 MHz, DMSO-*d*$_6$): δ = 9.08 (s, 1H, -N**H**), δ = 7.98 – 7.57 (m, 15H, -P**Ph**$_3$), δ = 7.09 (d, J = 7.2 Hz, 2H, benzene), δ = 6.99 (t, J = 7.6 Hz, 2H, benzene), δ = 6.86 (t, J = 7.3 Hz, 1H, benzene), δ = 4.80 (s, 1H, -C**H**-), δ = 4.18 – 3.98 (m, 2H, -C**H**$_2$-CH$_2$-CH$_2$-PPh$_3$), δ = 3.97 – 3.89 (m, 2H, -C**H**$_2$CH$_3$), δ = 3.47 – 3.35 (m, 2H, -CH$_2$-CH$_2$-C**H**$_2$-PPh$_3$), δ = 2.25 (s, 3H, -C**H**$_3$), δ = 2.22 (s, 3H, -C**H**$_3$), δ = 1.75 (m, J = 14.6, 7.3 Hz, 2H, -CH$_2$-C**H**$_2$-CH$_2$-), δ = 1.08 (t, J = 7.1 Hz, 3H, -CH$_2$C**H**$_3$). ^{13}C NMR (500 MHz, DMSO-*d*$_6$): δ = 14.09, 18.08, 18.19, 38.74, 58.92, 100.69, 102.18, 117.59, 118.27, 127.11, 127.73, 130.15, 130.25, 133.37, 133.45, 134.97, 145.06, 146.82, 148.15, 166.42, 166.83. ESI-MS (m/z) = 604.6 [M]$^+$.

1.4.3. (3-((5-(ethoxycarbonyl)-2,6-dimethyl-4-(thiophen-2-yl)-1,4-dihydropyridine-3-carbonyl)oxy)propyl)triphenylphosphonium (3)

Compound **3** was prepared using the general method described for the preparation of **1-3** from **8c** (307 mg, 1.0 mmol) to give **3** as a yellow powder (470 mg, yield=77%); ^{1}H NMR (500 MHz, DMSO-*d*$_6$): δ = 9.33 (s, 1H, -N**H**), 7.80 (dq, J = 19.1, 7.4 Hz, 15H, -P**Ph**$_3$), 7.04 (d, J = 6.0 Hz, 1H, thiophene), 6.68 – 6.61 (m, 1H, thiophene), 6.58 (d, J = 3.3 Hz, 1H, thiophene), 5.13 (s, 1H, -C**H**-), 4.15 (dd, J = 38.2, 33.0 Hz, 2H, -C**H**$_2$-CH$_2$-CH$_2$-PPh$_3$), 4.04 – 3.95 (m, 2H, -C**H**$_2$CH$_3$), 3.40

(t, J = 13.2 Hz, 2H, -CH_2-CH_2-C**H**$_2$-PPh_3), 2.26 (s, 3H, -C**H**$_3$), 2.23 (s, 3H, -C**H**$_3$), 1.87 – 1.75 (m, 2H, -CH_2-C**H**$_2$-CH_2-), 1.12 (t, J = 7.1 Hz, 3H, -CH_2C**H**$_3$). ^{13}C NMR (500 MHz, DMSO-d_6): δ = 14.97, 18.84, 18.88, 22.35, 34.5, 59.90, 63.01, 63.16, 101.00, 102.40, 118.40, 119.09, 123.13, 124.12, 127.07, 130.96, 131.06, 134.17, 134.25, 135.74, 135.76, 146.50, 148.07, 152.70, 166.97, 167.36. ESI-MS (m/z) = 610.5 $[M]^+$

2. Biological Evaluation

2.1. Cell Toxicity

Cell toxicity was also measured by NR assay. Cells were seeded into 96-well culture plates, culture medium (with Mito-DHPs) was removed after 48 hours. Cells were washed with PBS and 100 µl NR medium was added to all wells. Plates were incubated at 37°C under a humidified atmosphere with 5% CO_2 for 2 h. NR medium was removed and wells were washed gently with PBS, and 150 µl desorb solution was used to extract NR from living cells. Plates were gently agitated for 5 min and the optical absorbance was recorded at 540 nm using an Infinite F200 multimode plate reader.

2.2. Cell Survival

Cell survival was evaluated by the Neutral Red (NR) assay 24 h after radiation. Cells were washed with PBS three times and 100 µL NR medium (0.05 g/mL) was added to all wells. Plates were incubated at 37°C for 2 h. NR medium was removed and wells were washed gently with PBS three times, and 150 µl of desorb solution (glacial acetic acid: ethanol: water = 1: 50: 49) was used to extract NR from living cells. Plates were gently agitated for 5 min and the optical absorbance was recorded at 540 nm using an Infinite F200 multimode plate reader.

3. *Biological Results*

3.1. Cell Toxicity of Mito-DHPs

As shown in Figure S1, the Mito-DHP1 displayed minimum toxicity to CHO cells after 48 h treatment, which no cell damage or morphological abnormalities were observed at the highest dosage of 10^{-4} mol/L. Mito-DHP2 and Mito-DHP3 were well tolerated by cells in the range of 10^{-8}~10^{-5} mol/L. However, cell viability respectively decreased to 52.67% and 23.54% for Mito-DHP2 and Mito-DHP3 at the high dose of 10^{-4} mol/L. As for HeLa cells (Figure S2), Mito-DHP1 promoted cell growth dramatically at doses from 10^{-8} to 10^{-5} mol/L, among which 10^{-6} mol/L is the most outstanding dosage with cell viability up to 119.87%. Mito-DHP2 and Mito-DHP3 began to show dramatic toxicity to HeLa cells at 10^{-5} mol/L, when the cell viability was 58.08% and 64.27%. However, all the three compounds showed significant toxicity to HeLa cells at the highest dosage (10^{-4} mol/L), and cell viability reduced to 71.05, 23.78, and 22.95% under the treatment of Mito-DHP1, Mito-DHP2, and Mito-DHP3, respectively.

3.2. Cell Survival Following Radiation

To investigate whether the compounds could protect cells from radiation and to determine an appropriate dose for administration, five doses (10^{-4} to 10^{-8} mol/L) were tested for all three compounds and cell survival was monitored by measuring their ability to absorb Neutral Red (NR) dye (Figure S3, S4). For CHO cells, all doses of Mito-DHP1 protected cells from ionizing radiation and increased cell viability, but both Mito-DHP2 and Mito-DHP3 exhibited toxicity at a dose of 10^{-4} mol/L, resulting in cell death. In contrast, Mito-DHP1 did not exhibit any toxicity at the highest dose. Cell survival was highest for all compounds at a dose of 10^{-6} mol/L, which was also in consistent with results from HeLa cells. Therefore, 10^{-6} mol/L was considered as the optimum dosage.

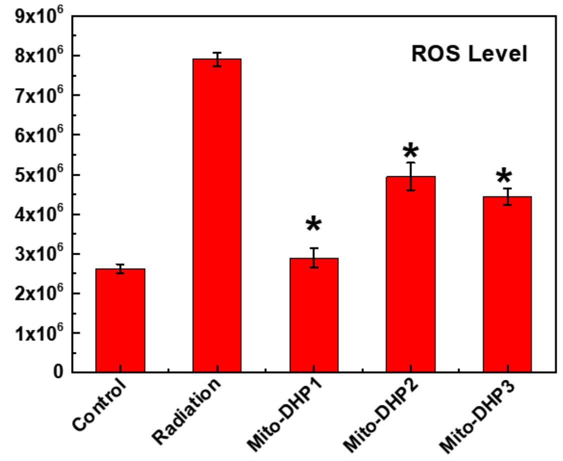

Figure S1. ROS level in Mito-DHP-treated and untreated HeLa cells at 24 h after radiation. (*p <0.05 compared with radiation group).

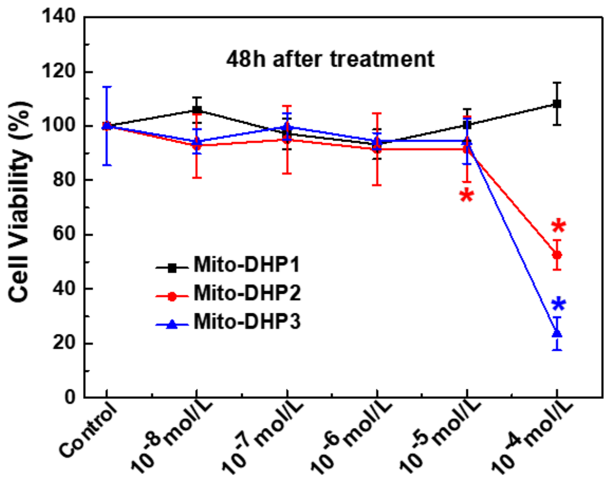

Figure S2. Cell toxicity measured by NR assay at 48 h after treatment. Mito-DHP1, Mito-DHP2 and Mito-DHP3 were tested at doses of 0, 10^{-8}, 10^{-7}, 10^{-6}, 10^{-5}, 10^{-4} mol/L. (*p <0.05 compared with control group)

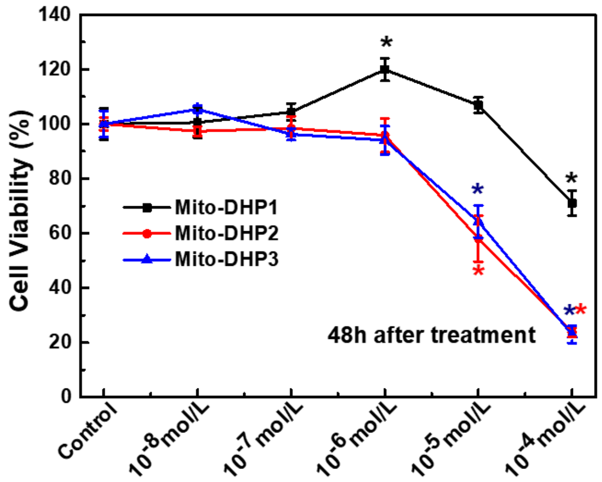

Figure S3. Toxicity experiment on HeLa cells line after 48 hours inbubation. Mito-DHP1, Mito-DHP2 and Mito-DHP3 were tested at doses of 0, 10^{-8}, 10^{-7}, 10^{-6}, 10^{-5}, 10^{-4} mol/L. (*p <0.05 compared with control group)

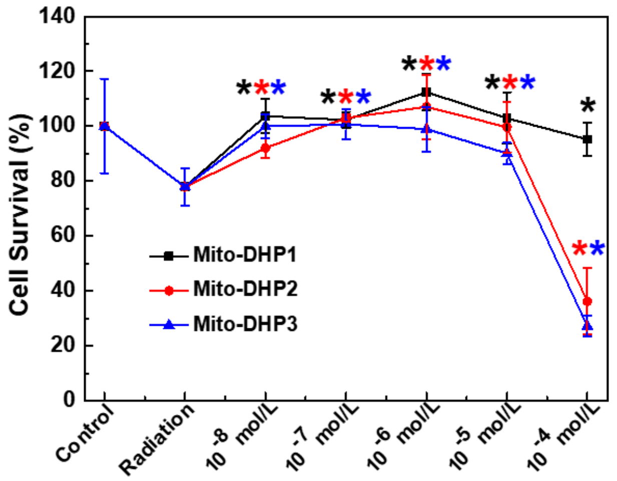

Figure S4. NR assay evaluation of CHO-K1 cells survival at 24 h after 3Gy radiation. Mito-DHP1, Mito-DHP2 and Mito-DHP3 were tested at a dose of 0, 10^{-8}, 10^{-7}, 10^{-6}, 10^{-5}, 10^{-4} mol/L. Control group were irradiated but not incubated with Mito-DHP compounds. (*p <0.05 compared with radiation group)

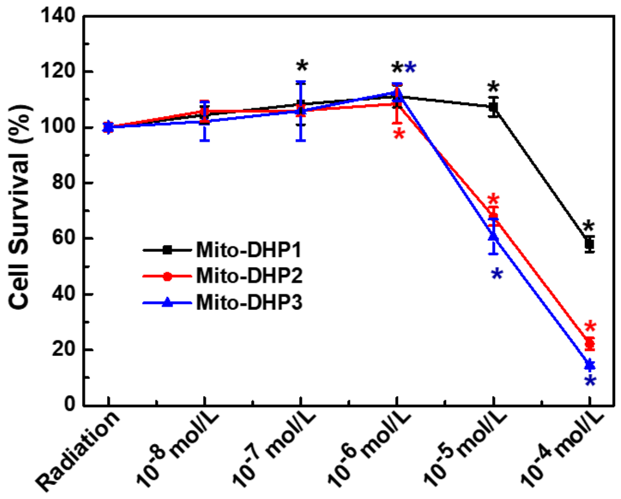

Figure S5. Evaluation of HeLa cells survival at 24 h after radiation. Mito-DHP1, Mito-DHP2 and Mito-DHP3 were tested at doses of 0, 10^{-8}, 10^{-7}, 10^{-6}, 10^{-5}, 10^{-4} mol/L 30 minutes before radiation. (*p <0.05 compared with radiation group)
